# Supplementary material for: Beyond kin killing: Dickeya-derived phage-tail-like bacteriocin P2D1 targets phylogenetically distant Pseudomonas spp
Source: ISME Commun. 2026 Jan 19;6(1):ycag012. doi: 10.1093/ismeco/ycag012 (PMC12904277; doi:10.1093/ismeco/ycag012)
Supplement: SupplementaryDataset1_ycag012 [file supplementarydataset1_ycag012.docx]

# Supplementary Information

*to a research article*

**Beyond kin killing: *Dickeya*-derived phage-tail like bacteriocin P2D1 targets phylogenetically distant *Pseudomonas* spp.**

Marcin Borowicz ^a^, Jan Styn ^a, #^, Kacper Tomasik ^a, #^, Łukasz Rąbalski ^b^, Magdalena Narajczyk ^c^, Erwan Gueguen ^d^, Sylwia Jafra ^e^, Julie Baltenneck ^d^, Dorota M. Krzyżanowska ^e,^*, Robert Czajkowski ^a,^ *

^a^ Laboratory of Biologically Active Compounds, Intercollegiate Faculty of Biotechnology of the University of Gdańsk and Medical University of Gdańsk, University of Gdańsk, ul. A. Abrahama 58, 80-307 Gdańsk, Poland;

^b^ Laboratory of Recombinant Vaccines, Intercollegiate Faculty of Biotechnology UG and MUG, University of Gdańsk, A. Abrahama 58, 80-307 Gdańsk, Poland

^c^ Bioimaging Laboratory, Faculty of Biology, University of Gdańsk, Gdańsk, Poland

^d^ University of Lyon, Université Lyon 1, INSA de Lyon, CNRS UMR 5240 Microbiologie Adaptation et Pathogénie, 69622 Villeurbanne, France

^e^ Laboratory of Plant Microbiology, Intercollegiate Faculty of Biotechnology of the University of Gdańsk and Medical University of Gdańsk, University of Gdańsk, A. Abrahama 58, 80-307 Gdańsk, Poland;

^#^ These authors contributed equally to this work

***** Correspondence: robert.czajkowski@ug.edu.pl; phone: 0048 58 523 6333; dorota.krzyzanowska@ug.edu.pl; phone: 0048 58 523 6316

# Supplementary Tables

## **Supplementary Table S1 Plasmids used in this study**

| **Plasmids** | **Description** | **Source** |
| --- | --- | --- |
| pRE112 | Suicide vector for allelic exchange, Cm^R^, *sacB*, *oriT* RP4, *ori*R6K | [[1](#_ENREF_1)] |
| pEGL332 | Amp^R^, pSC101 ori, lacZp expression vector, mobilizable | [[2](#_ENREF_2)] |
| pSJG | pEGL332-∆*Dda3937_03810-*∆*Dda3937_03811,* Amp^R^; | This study |

## Supplementary Table S2 Oligonucleotides used in this study

| **Oligonucleotide** | **Sequence (5’-3’)** | **Application** |
| --- | --- | --- |
| L6 | gttttcccagtcacgac | Verification of cloning in the MCS of pEGL332 |
| L7 | caggaaacagctatgacc |  |
| L762 | gttattggtgcccttaaacg | Verification of correct cloning into pRE112 |
| L763 | gcatccaacgccattcatgg |  |
| L1700 | aactgcatgaattcccgggagagctcgtgacggatgccaaaacgcag | Amplification of the upstream and downstream 0.5-kb DNA fragments of *D. dadantii Dda3937_03810* and their cloning into suicide vector pRE112, in order to obtain a deletion mutant in the respective locus |
| L1701 | ctccttagctgttatcactcatcaagaatgtctcctg |  |
| L1702 | ttgatgagtgataacagctaaggagcgacagac |  |
| L1703 | gatcccaagcttcttctagaggtaccggtcaacgccgttcactttc |  |
| L1705 | gatcccaagcttcttctagaggtaccagtcttgtccacttcgttgtag | Amplification of the upstream and downstream 0.5-kb DNA fragments of *D. dadantii Dda3937_03811* and their cloning into suicide vector pRE112, , to obtain a deletion mutant in the respective locus |
| L1706 | aactgcatgaattcccgggagagctcgaccggcatctccgccag |  |
| L1707 | gtcgttacagacccagtgccatgagtctgtcg |  |
| L1708 | ctcatggcactgggtctgtaacgaccgcttcatg |  |
| L1700 | aactgcatgaattcccgggagagctcgtgacggatgccaaaacgcag | Amplification of the upstream 0.5-kb DNA fragment of *Dda3937_03810,* and 0.5-kb downstream fragment of *Dda3937_03811* and their cloning into suicide vector pRE112 (to delete both loci/genes – a double mutant) |
| L1701 | ctccttagctgttatcactcatcaagaatgtctcctg |  |
| L1704 | ttgatgagtgataacagctaaggagggtctgtaacgaccgcttc |  |
| L1705 | gatcccaagcttcttctagaggtaccagtcttgtccacttcgttgtag |  |
| L1802 | ccgggctgcaggaattcgatatcaagcttgcgccaaacctctacagatg | Amplification of the region comprising *Dda3937_03810* and *Dda3937_03811* of *D. dadantii 3937* with its native promoter and cloning into pEGL332 to obtain complementation vector |
| L1803 | tcgaggtcgacggtatcgataagctttgaatggccagccgtagctgg |  |

# Supplementary Figures

## Supplementary Fig. S1 Morphology of P2D1 tailocin-susceptible *Pseudomonas* isolates grown on different media plates: TSA (A), M9 0.4% glucose (B), King's B (C), CAS agar for siderophore production (D), and crystal violet pectate (CVP) for the production of pectinases (E and F). Incubation time is indicated under each plate. On TSA, colonies of all tested *Pseudomonas* isolates exhibit a “slimy” morphology indicative of abundant extracellular matrix production. Images on King's B medium were taken under UV light (λ = 365 nm) to visualize pyoverdine fluorescence, characteristic of fluorescent pseudomonads. Where present, cavities on CVP medium are marked by arrows. *D. dadantii* 3937 was included as a reference for colony morphology and as a negative control for King’s B and a positive control on CVP.


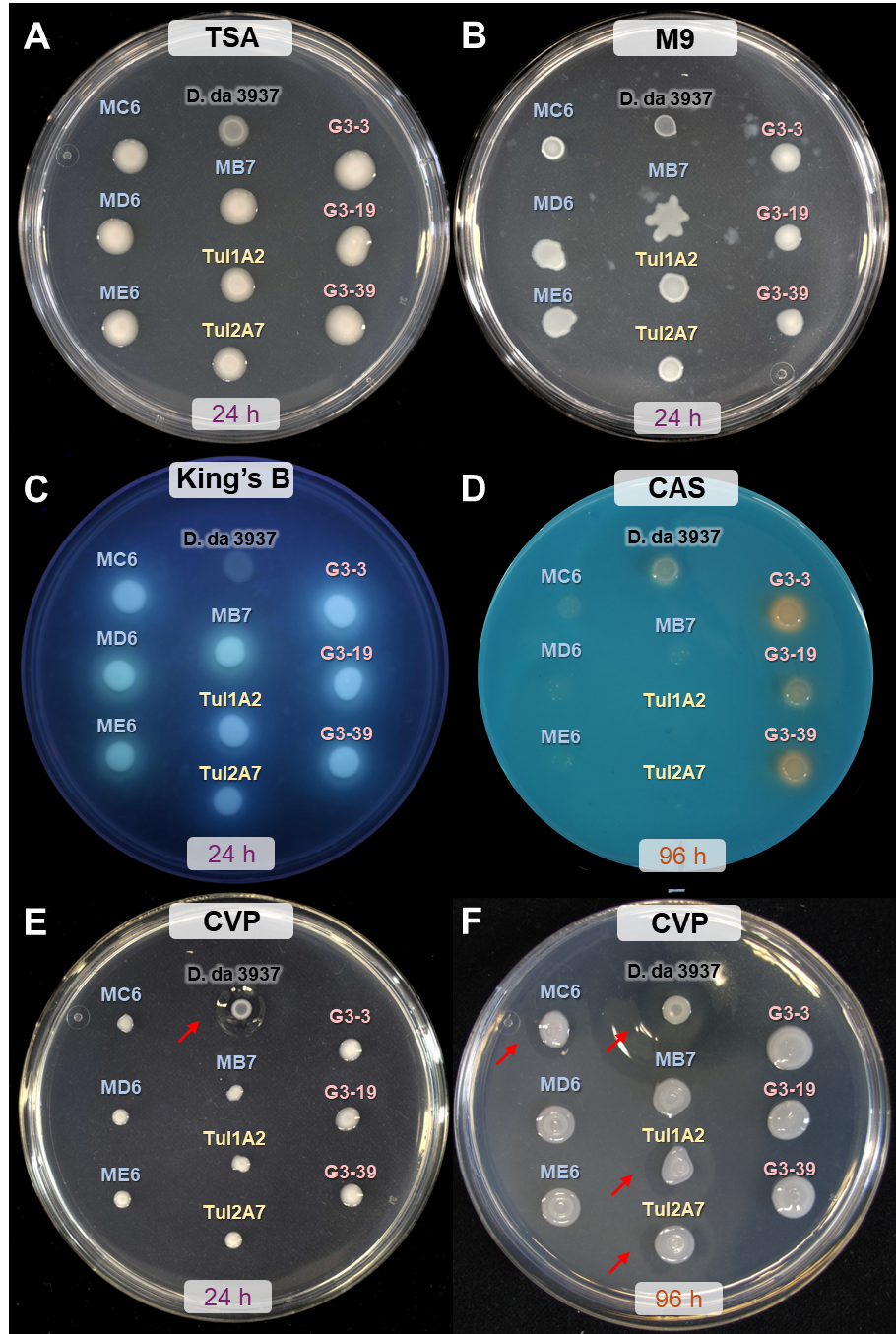


## Supplementary Fig. S2 TEM images of tailocins purified from mitomycin C-induced cultures of the wild-type *D. dadantii* 3937 (WT) and its mutants. Only tubes are visible in the sheath mutant Δ3810, while only sheath fragments are present in the tube mutant Δ3811. No complete tailocin particles were detected in the preparations from the double mutant Δ3810-11. Scale bar 50 nm.


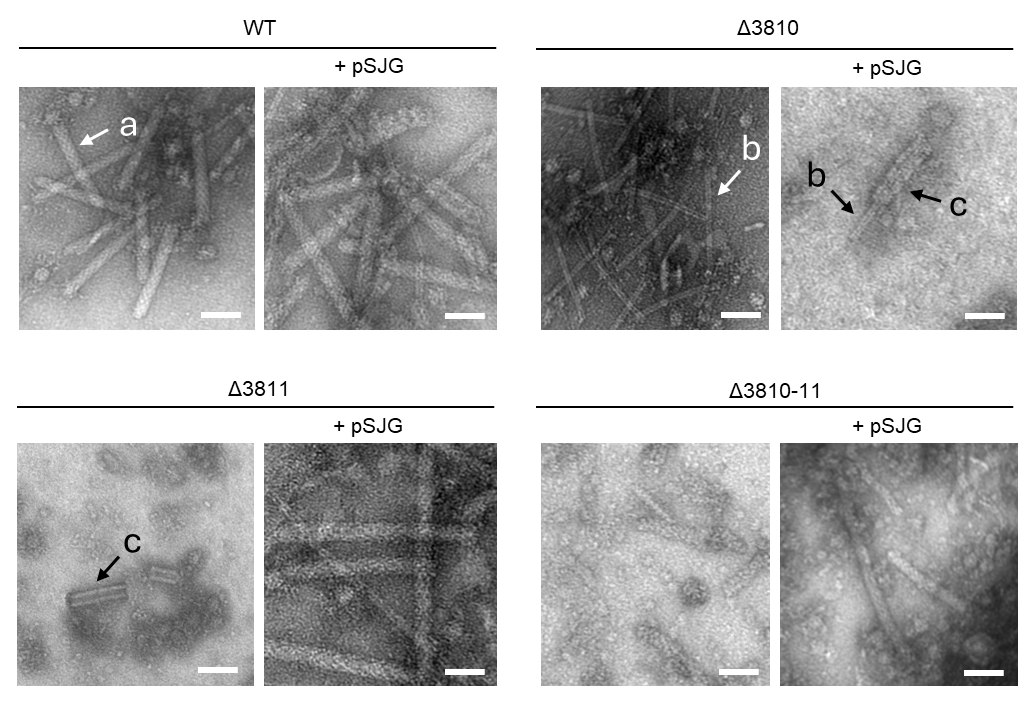


## Supplementary Fig. S3 Comparison of growth curves for *D. dadantii* 3937 and its tailocin-defficient mutants. Overnight cultures were prepared in (A) tryptic soy broth (TSB, Oxoid), (B) M9 minimal medium (MP Biomedicals) supplemented with 0.4% glucose (Sigma-Aldrich) at 28 °C with shaking at 120 rpm. These cultures were diluted in a 96-well plate by mixing 10 µL of the overnight culture with 190 µL of fresh TSB. Each strain was tested in 12 technical replicates, and the experiment was performed in triplicate. The plates were incubated at 28 °C with continuous shaking at 237 rpm in an Epoch 2 microplate reader (BioTek). Optical density (OD) at 600 nm was measured every 30 minutes over 24 hours. Results are shown as average. pSJG – complementation plasmid. TSB/M9 – control with sterile medium.


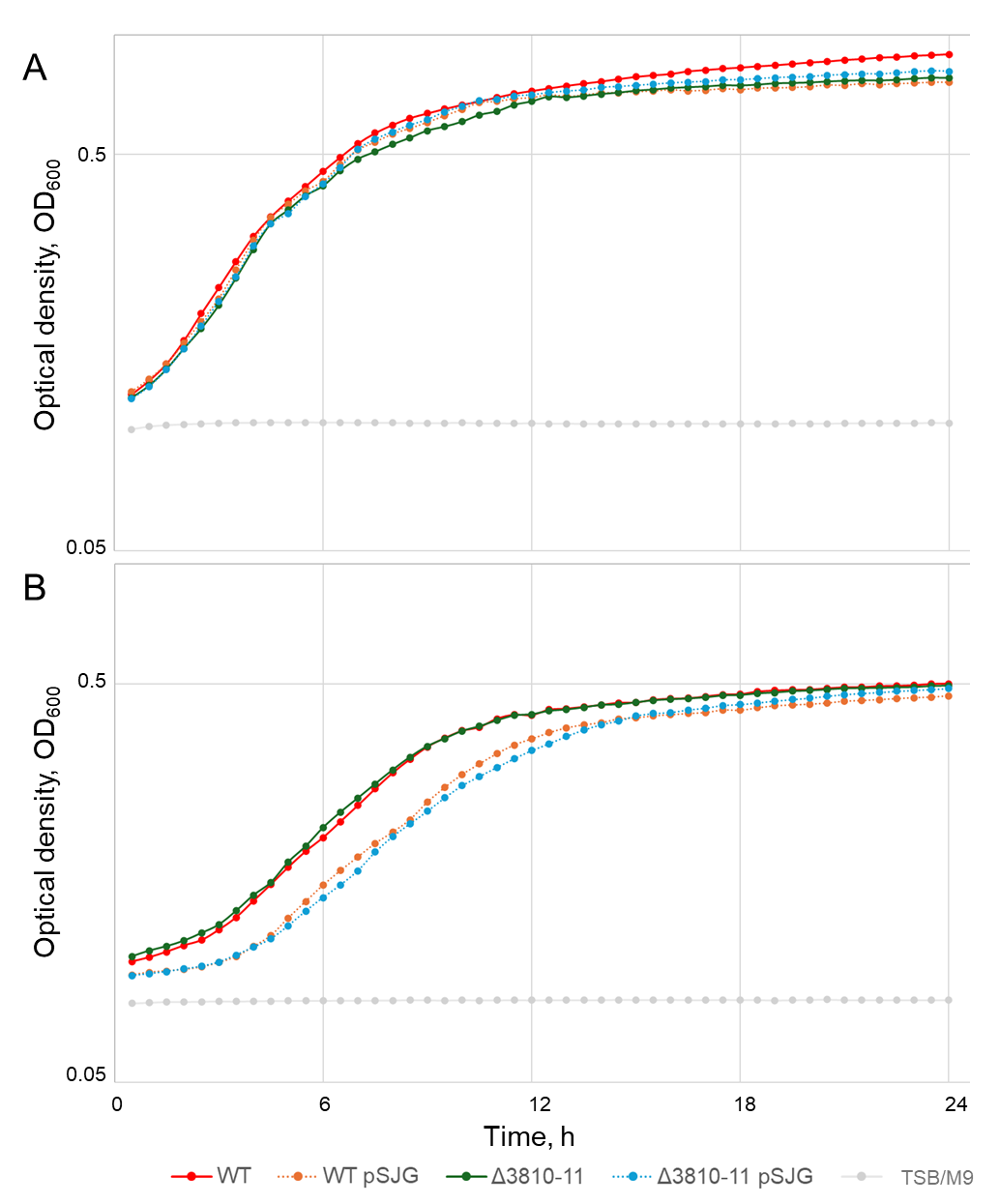


## Supplementary Fig. S4 Virulence on potato tubers in a whole-tuber injection assay. (A) Virulence of *D. dadantii* 3937 and its tailocin-deficient double mutant Δ3810-11; pSJG denotes the complementation plasmid. No significant differences were detected between the wild type and its derivatives (Mann–Whitney test, p > 0.05). (B) Virulence of P2D1 tailocin-susceptible *Pseudomonas* isolates. Significant differences were observed between the negative control (PBS) and *D. dadantii* 3937, as well as isolate MC6 (one-sample Wilcoxon test, p < 0.05; marked with asterisks). Data are shown as box plots: whiskers indicate minimum and maximum values, boxes the interquartile range, and horizontal lines the medians. Each point represents an individual potato tuber (n = 20).


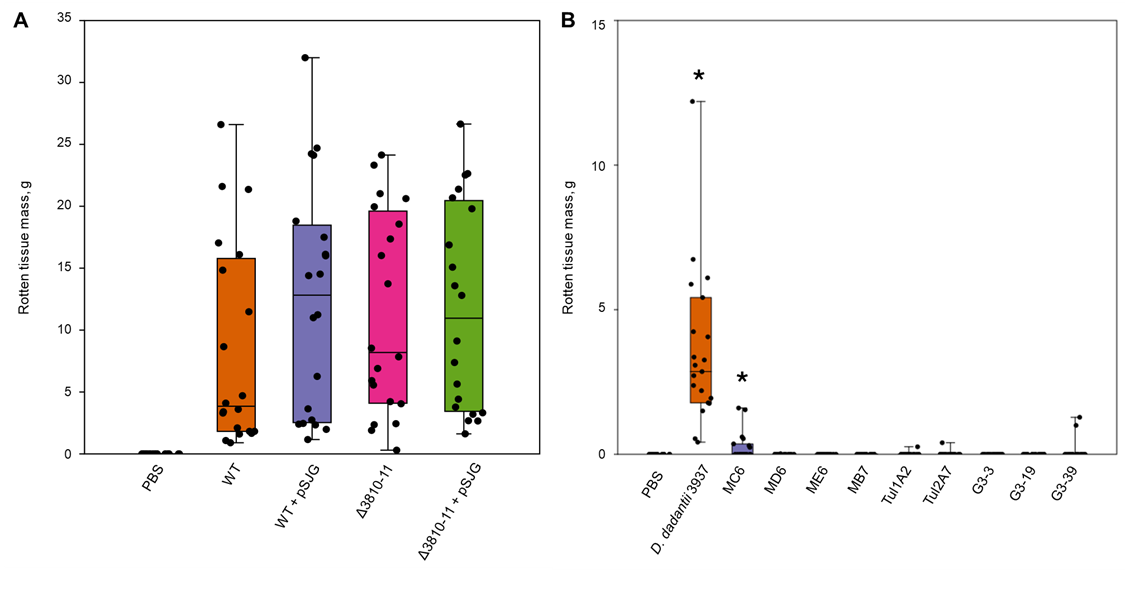


## Supplementary Fig. S5 Phenotypic profiling of *D. dadantii* 3937 and its tailocin-deficient mutant using BIOLOG assays.

(A) GEN III MicroPlate™ profiling (94 traits, including carbon utilization, chemical sensitivity, and physiological properties). (B) EcoPlate™ profiling (31 carbon-source utilization traits). Results were normalized to the negative control (positive reaction ≥2 × control) and averaged across three biological replicates. Data are presented as bar plots relative to the negative control baseline.

pSJG denotes the complementation plasmid.


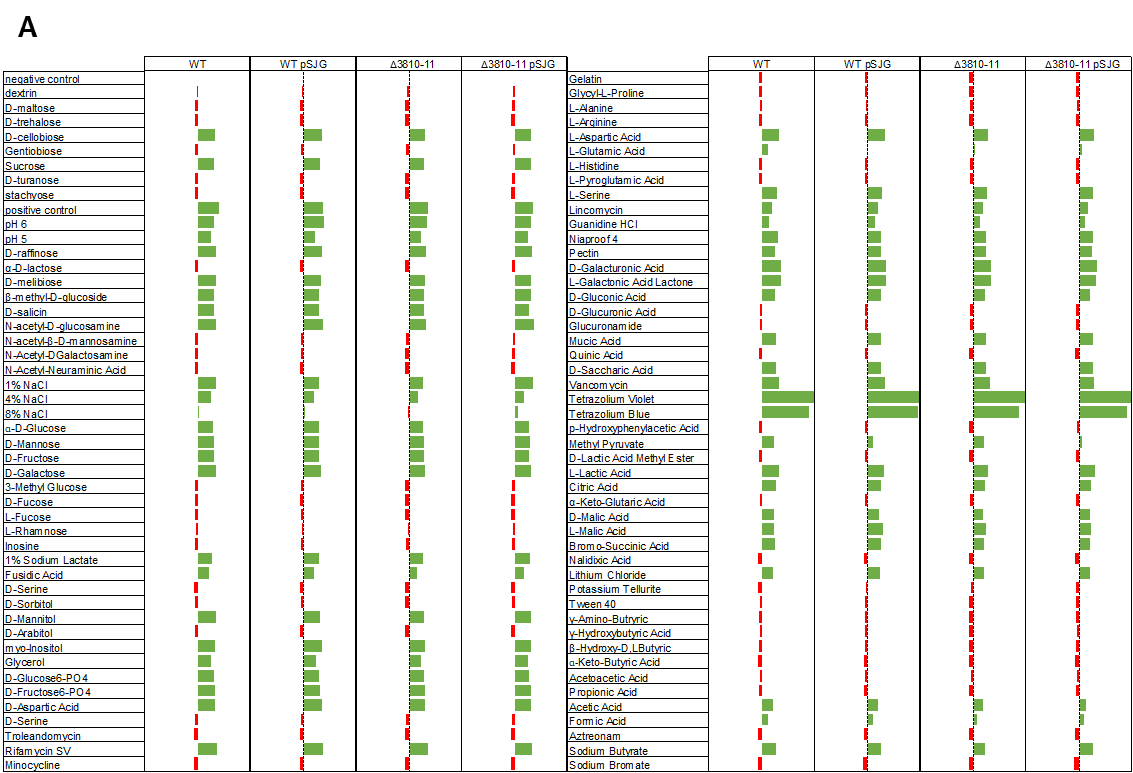


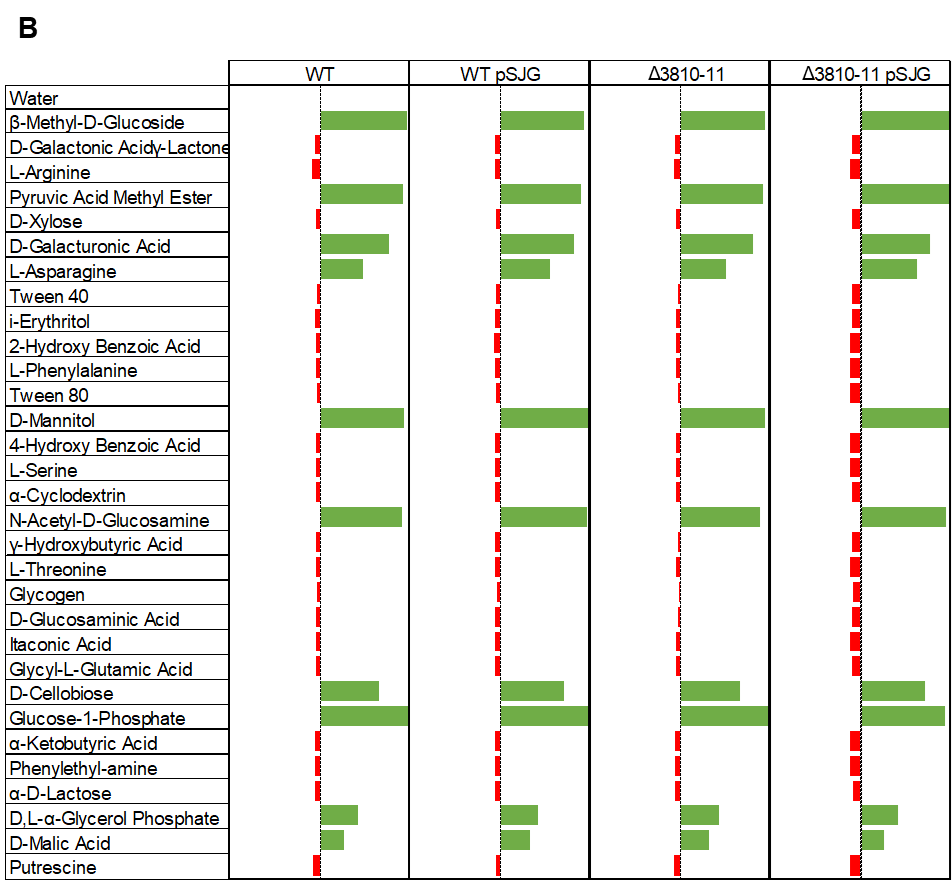


## Supplementary Fig. S6 Reciprocal antibiosis assay on TSA media plates between *D. dadantii*, its tailocin-deficient mutant, and environmental *Pseudomonas* strains. *D. dadantii* wild-type and the mutant were spotted onto the lawns of environmental *Pseudomonas* strains (A) and *vice versa* (B). Results from one of two independent experiments yielding identical outcomes are shown.


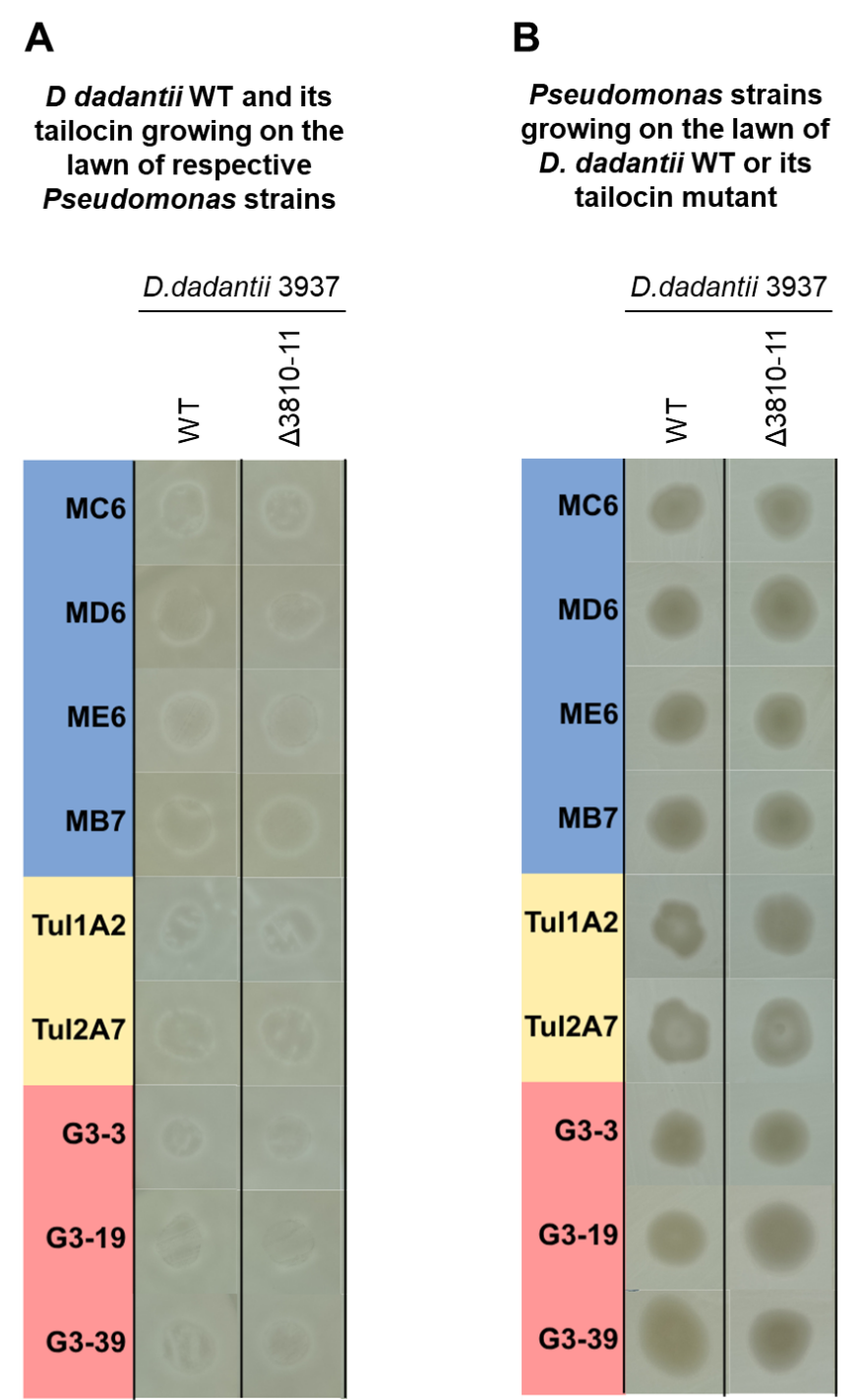


# Supplementary Datasets

## Supplementary Dataset 1 Isolates Taxonomical Placement Among *Pseudomonas* Type Strains Based On 16S rRNA Gene – Full Tree (pdf file).

## Supplementary Dataset 2 Average Nucleotide Identity Calculation (Excel file)

## Supplementary Dataset 3 Isolates Full Phenotype Profiles (Excel file)

## Supplementary Script 1 Hierarchical clustering and dendrogram visualization (txt file)

Uncategorized References

1. Brual T, Effantin G, Baltenneck J *et al.* A natural single nucleotide mutation in the small regulatory rna arcz of dickeya solani switches off the antimicrobial activities against yeast and bacteria. *PLOS Genetics*. 2023;**19**:e1010725 <https://doi.org/10.1371/journal.pgen.1010725>

2. Xia Y, Li K, Li J *et al.* T5 exonuclease-dependent assembly offers a low-cost method for efficient cloning and site-directed mutagenesis. *Nucleic Acids Res*. 2019;**47**:e15 <https://doi.org/10.1093/nar/gky1169>
